# Reply to Falconi et al.: Economic red herrings and resistance to new modeling hinder progress in assessing ethanol’s land use change

**DOI:** 10.1073/pnas.2216091119

**Published:** 2022-12-13

**Authors:** Tyler J. Lark, Nathan P. Hendricks, Aaron Smith, Nicholas Pates, Seth A. Spawn-Lee, Matthew Bougie, Eric G. Booth, Christopher J. Kucharik, Holly K. Gibbs

**Affiliations:** ^a^Nelson Institute for Environmental Studies, University of Wisconsin-Madison, Madison, WI 53726; ^b^Department of Energy (DOE) Great Lakes Bioenergy Research Center, University of Wisconsin-Madison, Madison, WI 53726; ^c^Department of Agricultural Economics, Kansas State University, Manhattan, KS 66506; ^d^Department of Agricultural and Resource Economics, University of California-Davis, Davis, CA 95616; ^e^Department of Agricultural Economics, University of Kentucky, Lexington, KY 40546; ^f^Department of Geography, University of Wisconsin-Madison, Madison, WI 53726; ^g^Department of Agronomy, University of Wisconsin-Madison, Madison, WI 53706; ^h^Department of Civil and Environmental Engineering, University of Wisconsin-Madison, Madison, WI 53706

Falconi et al. (1) question our assessment of corn ethanol’s domestic land use change (2) (LUC) based on the corn price analysis and contradictions with their preferred modeling studies. We reaffirm the validity of our price analysis and attest that innovative empirical methods can advance the science and ground our collective understanding of the US Renewable Fuel Standard’s (RFS) environmental impacts.

## Tangential Economic Observations Do Not Nullify the Price Impacts of the RFS

**Fig. 1.**
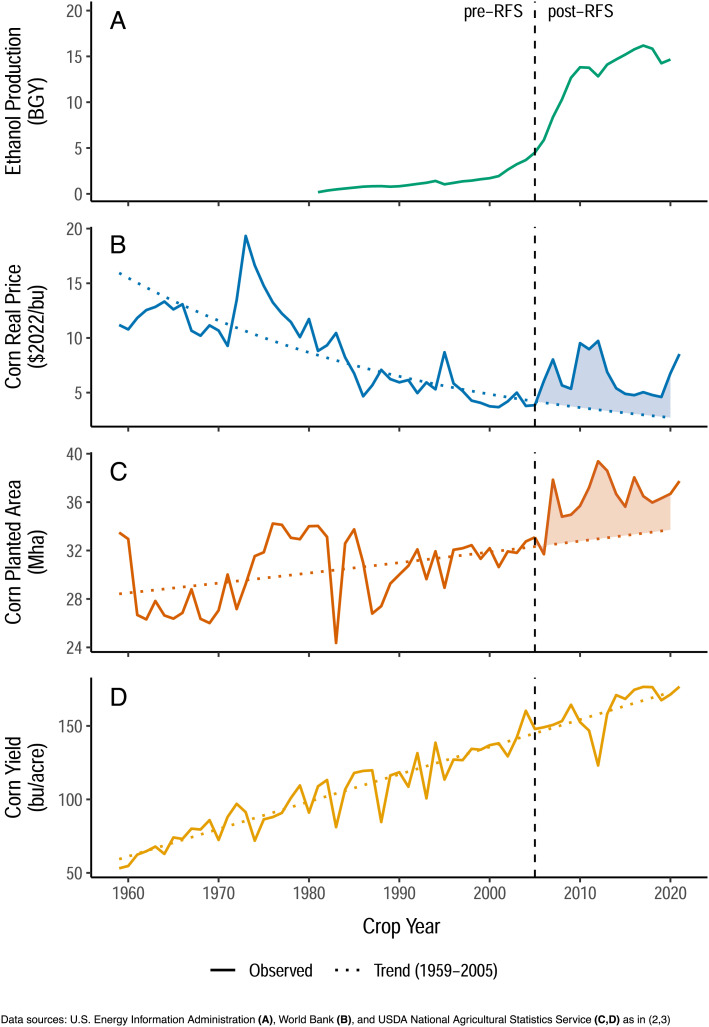
Inflation-adjusted corn prices and corn planted area have remained above their pre-RFS trends every year since the RFS, despite continued increases in yield.

Observations like those by Falconi et al. about correlations among corn prices, corn use for ethanol, and crude oil prices are correct but irrelevant to the effects of the RFS on crop prices. Around the time of the RFS, ethanol transitioned to a major user of corn and the price and planted area of corn have remained above their pre-RFS trends every year since then despite continued yield improvements (Fig. 1). Moreover, our model of the price impact does not simply compare prices before and after the RFS. Instead, we model the drivers of corn prices and compare the actual prices of corn to a counterfactual scenario in which there is no RFS but everything else remains the same (see Fig. 1 in ref. 2). Regarding the time period of analysis, applying an alternative method that is not specific to any particular post-RFS year or period produces an identical estimate for the policy’s price impact (2, 3).

The conditions in our price model are also realistic and representative. Our counterfactual prices exceed the operating costs of growing corn every year, which is the relevant information upon which farmers make planting decisions. Our price analysis and counterfactual simulation include changes only in corn, soybeans, and wheat prices because these are the dominant alternative crops. Nonetheless, our models of LUC in response to prices include all major crops produced in each region.

### Moving beyond Prior LUC Modeling Approaches Helps Advance Scientific Understanding.

**Fig. 2.**
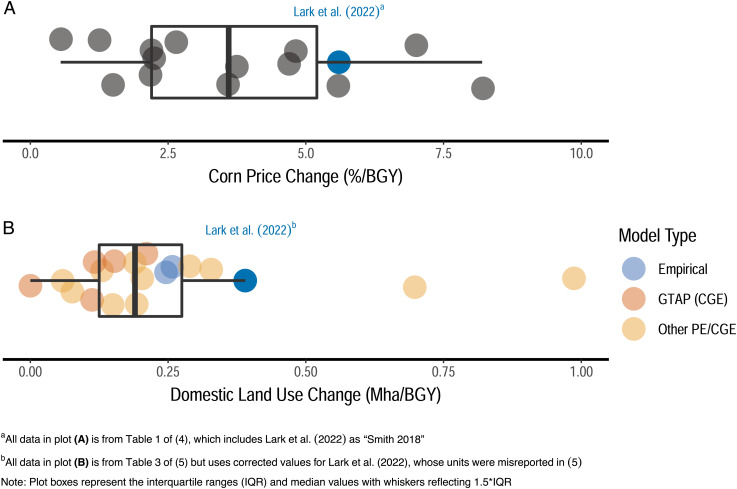
Normalized estimates for the (***A**)* price and (***B**)* LUC responses to increased corn ethanol demand from Lark et al. (2022) (2) are within the ranges of other studies cited by ref. 1 and others (4, 5).

Falconi et al. contend that differences between ours and previous approaches *a priori* reduce the validity of our study; we believe departing from entrenched modeling frameworks was necessary to advance knowledge in this domain. Regardless, our results for both corn prices and LUC responses actually fall within the ranges of those cited by Falconi et al. and others (Fig. 2) (4, 5). The noted differences in carbon intensity estimates for expanded corn ethanol instead arise largely from variation in LUC emissions per unit area and likely stem in part from systematic underestimation bias in some of the selected previous works (for details, see refs. 6 and 7). Furthermore, all of the “independently developed” models and results cited by Falconi et al. rely on similar approaches, which use partial equilibrium (PE) or computable general equilibrium (CGE) simulations, and several are from a single industry-supported model (GTAP) for which subjective choices about land representation have been shown to materially reduce biofuel carbon intensity estimates (8). Broader perspectives show that there remains wide and persistent variation among studies with no convergence toward robust values (5, 9).

We appreciate the opportunity provided by Falconi et al. to emphasize the importance of using economically rigorous estimates of commodity crop prices and of staying open to new approaches that move beyond run-of-the-mill modeling of biofuels’ LUC.
